# Temporally distinct roles for tumor suppressor pathways in cell cycle arrest and cellular senescence in Cyclin D1-driven tumor

**DOI:** 10.1186/1476-4598-11-28

**Published:** 2012-05-01

**Authors:** Hasan Zalzali, Mohamad Harajly, Lina Abdul-Latif, Nader El-Chaar, Ghassan Dbaibo, Stephen X Skapek, Raya Saab

**Affiliations:** 1 Department of Pediatric and Adolescent Medicine, American University of Beirut, Beirut, Lebanon; 2 Children’s Cancer Center of Lebanon, American University of Beirut, Beirut, Lebanon; 3 Pediatric Hematology-Oncology, University of Chicago, Chicago, Illinois, USA

**Keywords:** p18Ink4c, Cyclin D1, Senescence, p53, Rb, Cdk2, Tumor, Reactive oxygen species

## Abstract

**Background:**

Cellular senescence represents a tumor suppressive response to a variety of aberrant and oncogenic insults. We have previously described a transgenic mouse model of Cyclin D1-driven senescence in pineal cells that opposes tumor progression. We now attempted to define the molecular mechanisms leading to p53 activation in this model, and to identify effectors of Cyclin D1-induced senescence.

**Results:**

Senescence evolved over a period of weeks, with initial hyperproliferation followed by cell cycle arrest due to ROS production leading to activation of a DNA damage response and the p53 pathway. Interestingly, cell cycle exit was associated with repression of the Cyclin-dependent kinase Cdk2. This was followed days later by formation of heterochromatin foci correlating with RB protein hypophosphorylation. In the absence of the Cdk4-inhibitor *p18Ink4c*, cell cycle exit was delayed but most cells eventually showed a senescent phenotype. However, tumors later arose from this premalignant, largely senescent lesion. We found that the p53 pathway was intact in tumors arising in a *p18Ink4c-/-* background, indicating that the two genes represent distinct tumor suppressor pathways. Upon tumor progression, both *p18Ink4c-/-* and *p53-/-* tumors showed increased Cdk2 expression. Inhibition of Cdk2 in cultured pre-tumorigenic and tumor cells of both backgrounds resulted in decreased proliferation and evidence of senescence.

**Conclusion:**

Our findings indicate that the p53 and the RB pathways play temporally distinct roles in senescence induction in Cyclin D1-expressing cells, and that Cdk2 inhibition plays a role in tumor suppression, and may be a useful therapeutic target.

## Introduction

Cellular senescence is a well-established tumor suppressor mechanism, activated in response to oncogenic signals, DNA damage, and telomere attrition among other pro-tumorigenic insults (reviewed in [1,2]). Mechanistic insight into oncogene-induced senescence has emerged over the past few years. While it is clear that both the p53 and the Rb tumor suppressors are involved [3-6], their relative importance seems to vary depending on the activating insult and cellular context [3,7-10]. Understanding the relative contributions of p53 and Rb to the induction and maintenance of senescence may have important implications, especially with development of targeted therapeutic agents.

Cyclin-dependent kinase 2 (Cdk2) was recently implicated in the senescence process, as Cdk2 loss was found to enhance senescence in Myc-induced tumors [11]. In addition, it was shown that Cdk2-dependent phosphorylation of Myc was necessary to bypass Ras-induced senescence [12]. This suggests that Cdk2 may act in senescence independently of its role in RB phosphorylation and cell cycle exit.

Here, we used a transgenic mouse model of premalignant Cyclin D1-driven pineal gland hyperplasia, to define the molecular mechanisms leading to p53 activation in response to Cyclin D1, and to identify effectors of Cyclin D1-induced senescence. The results shed light on the pattern of evolution of the senescence response in a premalignant lesion *in vivo*, and the differences in the contribution of the two major tumor suppressor pathways, p53 and RB. In addition, our findings suggest that Cdk2 inhibition may be a useful therapeutic approach, irrespective of the underlying genetic insult that led to senescence evasion.

## Results

**Figure 1:**
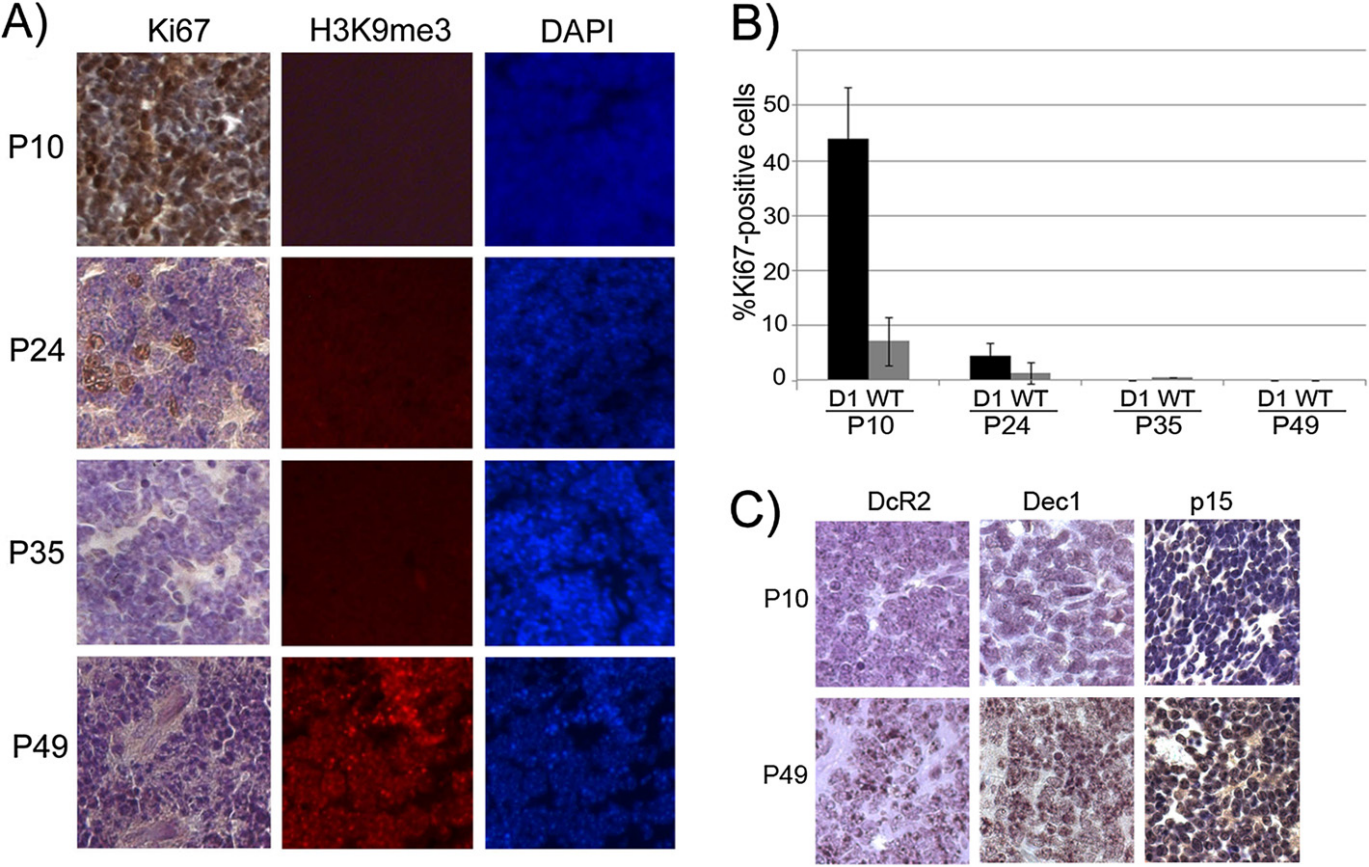
**Cyclin D1-induced senescence occurs over several weeks. A) **Ki67 staining of *Irbp-Cyclin D1 *pineal sections at the indicated ages (left); immunoflourescence staining for Histone 3 trimethylated at Lysine 9 (H3K9me3) in pineal sections at the indicated ages (middle), with corresponding DAPI staining (right). **B) **Quantitation of Ki67-positive cells as percent of total pineal cells at indicated ages. **C) **Immunostaining for Dec1, DcR2, and p15Ink4b, in Irbp-Cyclin D1 pineal glands at the indicated ages.

Cyclin D1-induced senescence* in vivo *occurs over a time frame of several weeks: We used the *Irbp-Cyclin D1* mouse in which the expression of Cyclin D1 in the pineal gland causes excessive proliferation that is limited by senescence. The net result is a hyperplastic but senescent pineal gland that does not progress into an invasive tumor unless either p53 or the Cdk4-inhibitor p18^Ink4c^ is lost [13]. We examined the temporal evolution of senescence and the contribution of the p53 and Rb tumor suppressor pathways to cell cycle exit *in vivo*. Histological studies of Cyclin D1-expressing pineal glands at various ages (post-natal day (P)10, P24, P35, and P49) showed that enhanced proliferation, measured by Ki67 immunostaining, was apparent at P10 but after this point it decreased such that essentially all cells had exited the cell cycle by P35 [Figure 1A, left panel, Quantitation in Figure 1B]. Cessation of proliferation preceded the formation of senescence-associated heterochromatin foci (SAHF); in fact, there was an unexpectedly long, two-week delay from P35 to P49 before SAHF were apparent [Figure 1A, middle and right panels]. Because SAHF have been observed in only a few mouse models of senescence [14-16], but not in other murine cells like MEFs [17], and because constitutive centromeric heterochromatin may show intense DAPI staining mimicking SAHF in normal murine cells [18,19], we verified that these foci were indeed only seen in Cyclin D1-expressing cells and not the wild-type counterparts at P49 Additional file 1: Figure S1A]. We could not detect positive staining for senescence-associated beta galactosidase (SABG), another marker of senescence [20]. However, we believe this must be a technical issue (detecting the enzyme activity necessitates freshly frozen tissue), because when the Cyclin D1-expressing pineal cells were grown *in vitro*, they showed features of senescence that included positive staining for SABG [see below, and Additional file 1: Figure S1B]. In addition, we evaluated the pineal cells for other known markers of senescence, such as Dec1, DcR2, and p15Ink4b [21]. We found that, concomitant with cell cycle exit and SAHF formation, all three markers of senescence were increased at P49 [Figure 1C]. We conclude that Cyclin D1-induced senescence in the pineal cells occurs by P49, and that cell cycle exit in this setting occurs many days prior to expression of *bona fide* markers of senescence.

**Figure 2:**
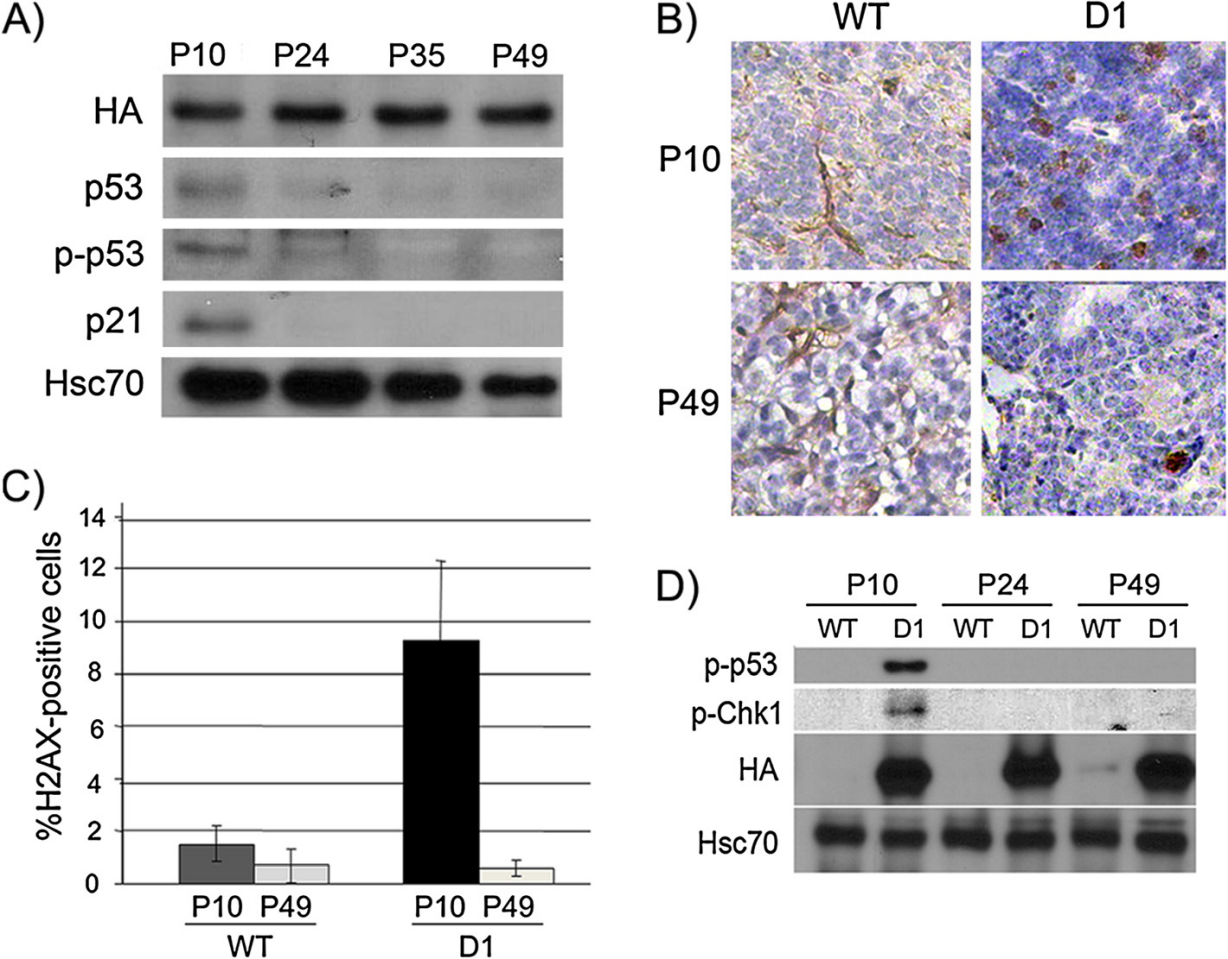
**Cyclin D1-induced cell cycle exit is associated with early p53 activation and a DNA damage response. A) **Western blotting for the indicated proteins in *Irbp-Cyclin D1 *pineal glands at the indicated ages. HA: HA epitope on the Cyclin D1 transgene. **B) **Representative immunostaining for γH2AX in *Irbp-Cyclin D1 *(D1) and *wild-type *(WT) pineal sections at the indicated ages. **C) **Quantitative analysis of γH2AX -positive pinealocytes in *wild-type *(WT) and *Irbp-Cyclin D1 *(D1) pineal sections at the indicated ages. Columns: mean of measurements from three to six separate mice; bars, SD. **D) **Western blotting for the indicated proteins in *wild-type *(WT) and *Irbp-Cyclin D1 *(D1) pineal glands at the indicated ages. HA: HA epitope on the Cyclin D1 transgene. Hsc70 is a loading control.

p53 activation occurs prior to cell cycle exit, and is mediated by a ROS-induced DNA damage response: To identify when p53 was engaged in tumor suppression, we evaluated cell lysates of pineal glands collected at different postnatal ages. We found that at P10, p53 protein expression was increased, as was its phosphorylation at Ser15/20 (a site phosphorylated by the DNA damage response) [Figure 2A]. The p53 target, p21^Cip1^, was also induced at that time-point [Figure 2A]. However, p53 activation did not persist once the cells had exited the cell cycle nor was it detectable at P49 when the cells displayed SAHF [Figure 2A].

We hypothesized that Cyclin D1 expression may be inducing p53 through activation of the DNA damage response (DDR), as reported for Ras-induced senescence (reviewed in [22]). Indeed, we found nuclear accumulation of phosphorylated histone H2AX (pH2AX) at P10, concomitant with p53 activation [Figure 2B, 2C]. In addition, Chk1 was phosphorylated concomitantly with phosphorylation of p53, further indicating that the DDR pathway was active in the transgenic pineal gland at this time, but not at later time-points [Figure 2D]. These findings reveal that deregulated Cyclin D1 enhances the DDR pathway and activates p53 while cells are proliferating, but ongoing DDR and active p53 are not needed after cells have undergone senescence.

**Figure 3:**
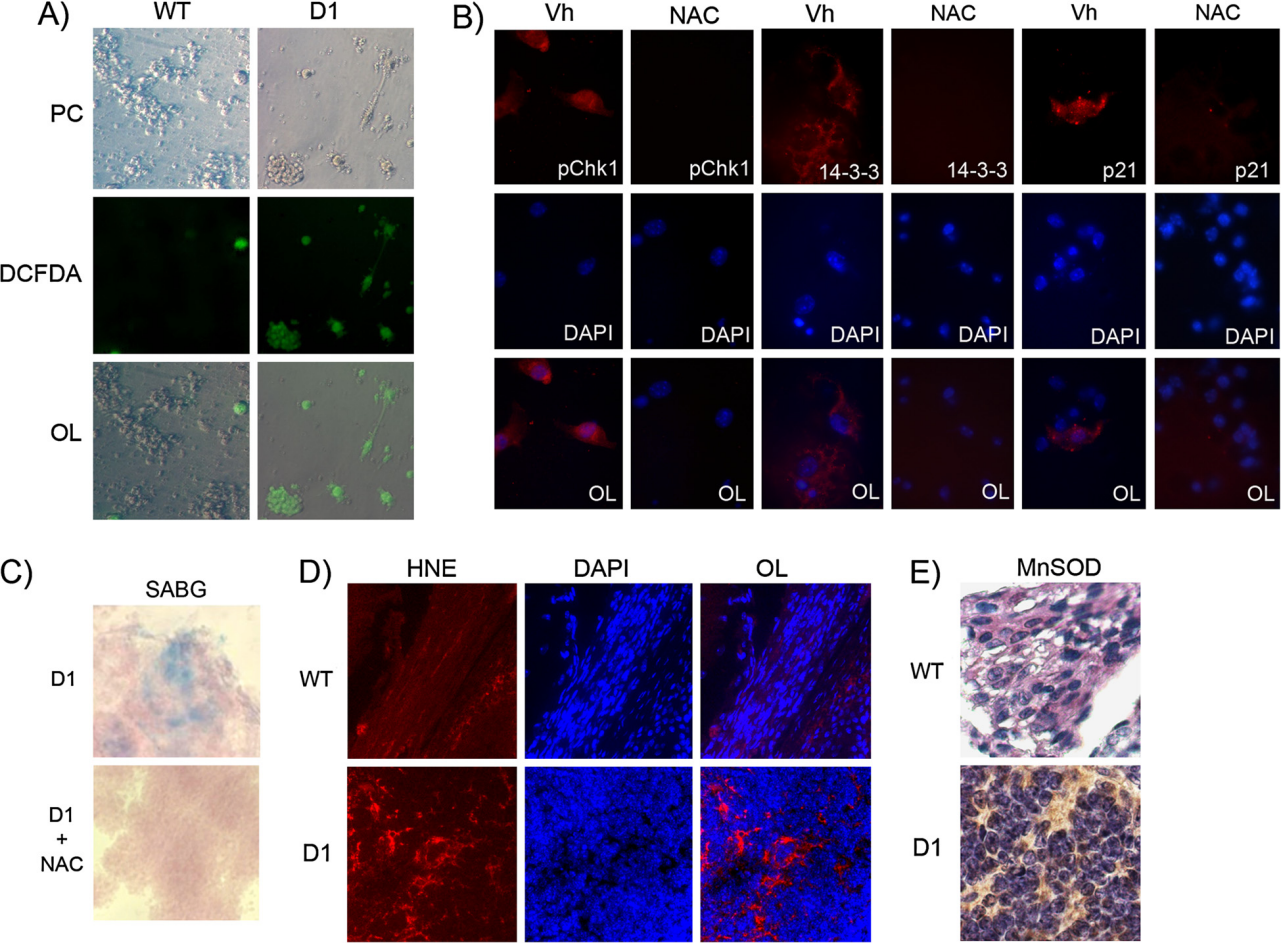
**Cyclin D1 expression leads to ROS accumulation, which results in a DNA damage response and senescence induction. A) **DCFDA assay in cultured pineal cells that are either wild-type (WT) or express Cyclin D1 (Cyclin D1). PC = phase contrast; OL = overlay. **B) **Immunofluorescence staining for the respective proteins in cultured Cyclin D1-expressing pineal cells (top panel), which have been treated with N-Acetyl Cysteine (NAC) or vehicle. The middle panel shows the respective DAPI-stained nuclei, and the lower panel shows the overlay (OL). **C) **Staining for senescence-associated beta galactosidase (SABG) in explanted Cyclin D1-expressing cells that have been treated with either NAC or vehicle, as indicated. **D) **Representative immunofluorescence staining for 4-hydroxynonenal (4HNE) in 10-day old *Irbp-Cyclin D1 *(D1) and wild-type (WT) pineal sections, as indicated. **E) **Representative immunostaining for the mitochondrial superoxide dismutase MnSOD in wild-type (WT) and *Irbp-Cyclin D1 *(D1) pineal sections, as indicated.

We next assessed whether reactive oxygen species (ROS) may be contributing to Cyclin D1-induced senescence in pineal cells, based on their role in Ras-induced senescence in cultured fibroblasts [23-25]. Using DCF-DA assay in explanted pineal cells, we found that ROS were indeed induced in response to Cyclin D1 expression, but not in wild-type pineal cells grown in the same conditions [Figure 3A]. We investigated whether ROS were responsible for activation of the DNA damage response in this setting. Indeed, Cyclin D1-induced ROS resulted in DNA damage foci marked by pH2AX, as well as the ROS-induced incorporation of oxidized dNTP, 8-Oxo-dGTP, into DNA Additional file 1: Figure S1D]. It also led to phosphorylation of Chk1, a component of the DNA damage response pathway [Figure 3, left panel], and to increased expression of two p53 pathway effector proteins, 14-3-3 and p21 [Figure 3B, middle and right panels]. Treating cells with the ROS scavenger N-Acetyl Cysteine (NAC) resulted in abrogation of DNA damage Additional file 1: Figure S1D], abrogation of DDR activation, and absence of p53 pathway activation [Figure 3B], as well as evasion of senescence [Figure 3C]. By staining for 4-hydroxy-nonenal (HNE), a marker of lipid oxidation, we found evidence of oxidative stress in pineal sections of *Irbp-Cyclin D1* mice, but not wild-type mice [Figure 3D], and there was also increased expression of the mitochondrial superoxide dismutase protein MnSOD [Figure 3E], which is induced by ROS stress *in vivo *[26]. Thus, from the above *in vitro* and *in vivo* evidence, we conclude that Cyclin D1 expression results in accumulation of ROS, which in turn leads to activation of the DDR and the p53 pathway, resulting in induction of senescence.

**Figure 4:**
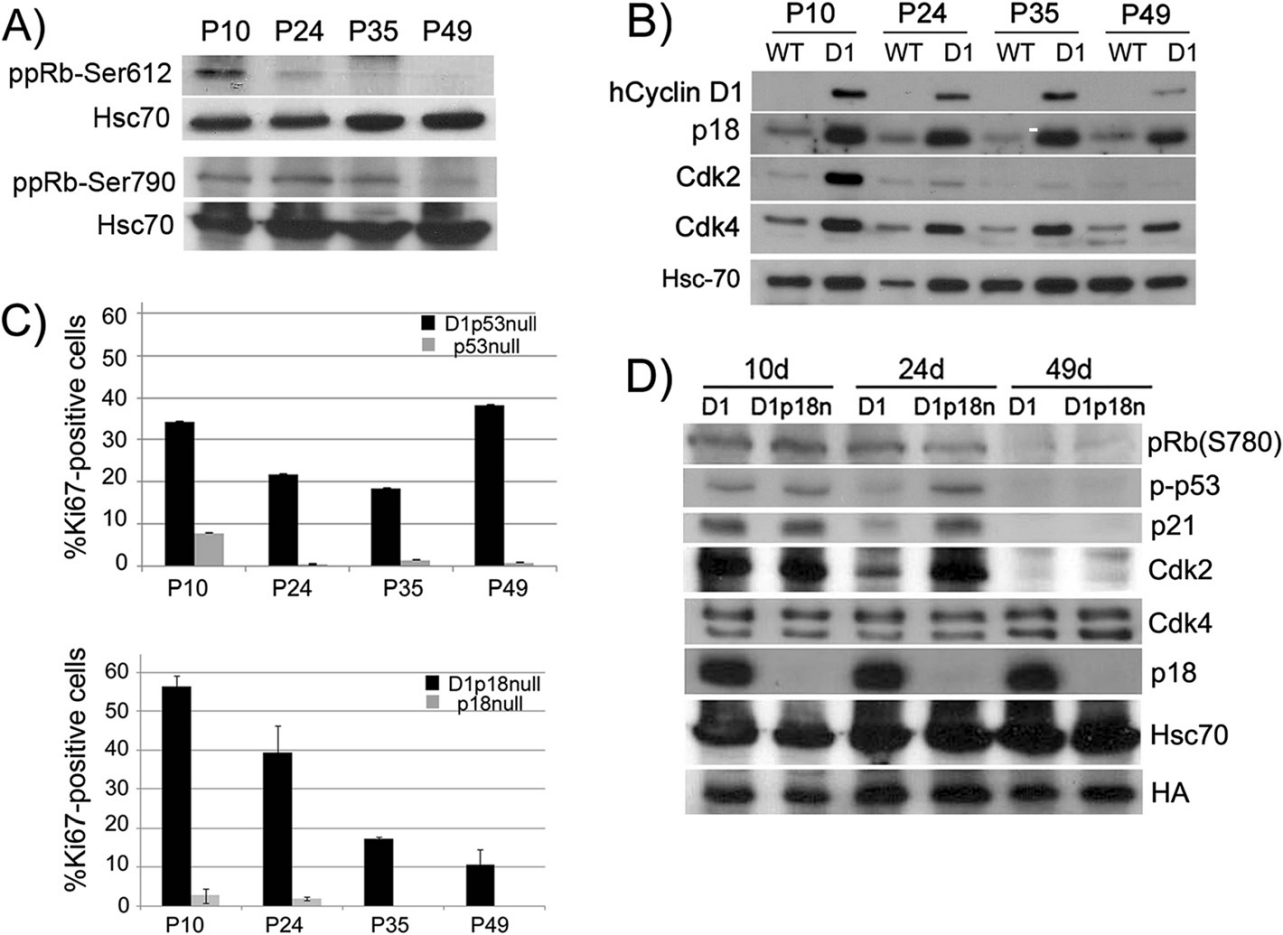
** *p18Ink4c *loss delays Cyclin D1-induced senescence. A) **and **B) **Western blotting for the indicated proteins in *wild-type *(WT) and *Irbp-Cyclin D1 *(D1) pineal glands at the indicated ages. Hsc70 is a loading control. **C) **Quantitative analysis of Ki67-positive pinealocytes in *Irbp-Cyclin D1, p53-/- *(D1p53n), and *Irbp-Cyclin D1, p18Ink4c-/- *(D1p18n) mice of the indicated ages, compared to *p53-/- *and *p18Ink4c-/- *controls, respectively. Columns: mean of measurements from three to six separate mice; bars, SD. **D) **Western blotting for the indicated proteins in *Irbp-Cyclin D1 *(D1) and *Irbp-Cyclin D1, p18Ink4c-/- *(D1p18n) pineal glands at the indicated ages. HA: epitope on the Cyclin D1 transgene.

RB pathway activation occurs after cell cycle exit and is associated with SAHF: To evaluate when the Rb pathway was engaged, we used western blotting to investigate the Cdk-dependent phosphorylation of Rb in pineal cell lysates. We investigated the status of Rb phosphorylation at Cdk4-dependent sites such as Ser790, and at Cdk2-dependent sites such as Ser612 [Figure 4A]. We found that Rb was phosphorylated at Cdk2-dependent sites (Ser612) at P10, when cells were proliferating, but decreased after cells exited the cell cycle by P24 [Figure 4A]. In contrast, Rb was phosphorylated at Cdk4-dependent sites (Ser790) from P10 through P35, even though most cells had ceased to proliferate by P24 [Figure 4A]. Rb phosphorylation at Cdk4-dependent sites was reversed at P49 as SAHF formed [Figure 4A].

To understand the mechanism of Rb activation, we investigated the expression of the Cdk-inhibitors p16Ink4a, p15Ink4b, p18Ink4c, and p27Kip1 [27-30]. There was increased expression of p18^Ink4c^ at all time points [Figure 3B], an increase in p15^Ink4b^ expression at P49 [see Figure 1D, but no changes in expression of p16^Ink4a^ and p27^Kip1^ (not shown). We also evaluated the expression of Cdk4 and Cdk2, especially since Cdk2 inhibition was recently found to be important for Myc-induced senescence [11]. We observed a modest decrease in Cdk4 expression from P10 through P49 [Figure 3B], but interestingly we found that Cdk2 expression was markedly reduced from P10 to P24 [Figure 3B], coincident with the timing of cell proliferation arrest and loss of Rb phosphorylation at Cdk2-specific sites. We conclude that Cdk2 repression correlates most closely with the initial proliferation arrest; and that diminished Cdk4-dependent Rb phosphorylation occurs at a later time-point and correlates with formation of SAHF.

p18^Ink4c^loss delays p53-dependent cell cycle exit: Because of the observed increase in expression of p18^Ink4c^, we used a genetic approach to evaluate its role in Cyclin D1-induced senescence. In contrast to what occurs without p53 – where cell proliferation only slightly decreases from P10 to P35 and then increases as invasive tumor progresses [Figure 4C, top panel; and [13]], proliferation decreased from P10 through P49 without *p18Ink4c* [Figure 4C, bottom panel], but exceeded that in the *Irbp-Cyclin D1* cells [Figure 4C, bottom panel, compare with Figure 1B].

Notably, p53 activation measured by phosphorylation at Ser15/20, and expression of the p53-target p21^Cip1^, persisted until P24 in *Irbp-Cyclin D1, p18Ink4c -/-* cells [Figure 4D], correlating with the prolonged cellular proliferation. Further, Cdk2 expression persisted until P24 in *Irbp-Cyclin D1, p18Ink4c -/-* cells [Figure 4]. These findings indicate that *p18Ink4c* loss delayed but did not prevent p53-dependent events leading to cell cycle exit.

Interestingly, loss of Cdk4-dependent Rb phosphorylation still occurred in the absence of *p18Ink4c*, again correlating with the appearance of SAHF [Figure 4D]. In fact, the majority of *Irbp-Cyclin D1, p18Ink4c -/-* cells displayed SAHF by P49 Additional file 2: Figure S2A], whereas SAHF never formed in *Irbp-Cyclin D1, p53 -/-* cells [13]. In addition to SAHF, the senescence markers Dec1 and DcR2 were also expressed in *Irbp-Cyclin D1, p18Ink4c -/-* cells at P49 Additional file 1: Figure S2B]. Findings were similar *in vitro* using pineal cells explanted from P10 animals and cultivated for 10-20 days: Explanted *Irbp-Cyclin D1* cells showed evidence of senescence, including loss of proliferation (measured by BrdU incorporation), and positive staining for SABG, by 10 days in culture Additional file 1: Figure S1B, 1C], while the *Irbp-Cyclin D1, p53 -/-* cells continued to proliferate and did not senesce Additional file 1: Figure S1B, bottom]. In contrast, the *Irbp-Cyclin D1, p18Ink4c -/-* cells did show evidence of senescence, but it was delayed until close to 20 days in culture Additional file 2: Figure S2C]. We conclude that p18^Ink4c^ slowed proliferation but was not essential for most Cyclin D1 expressing cells to cease proliferating and become senescent.

**Figure 5:**
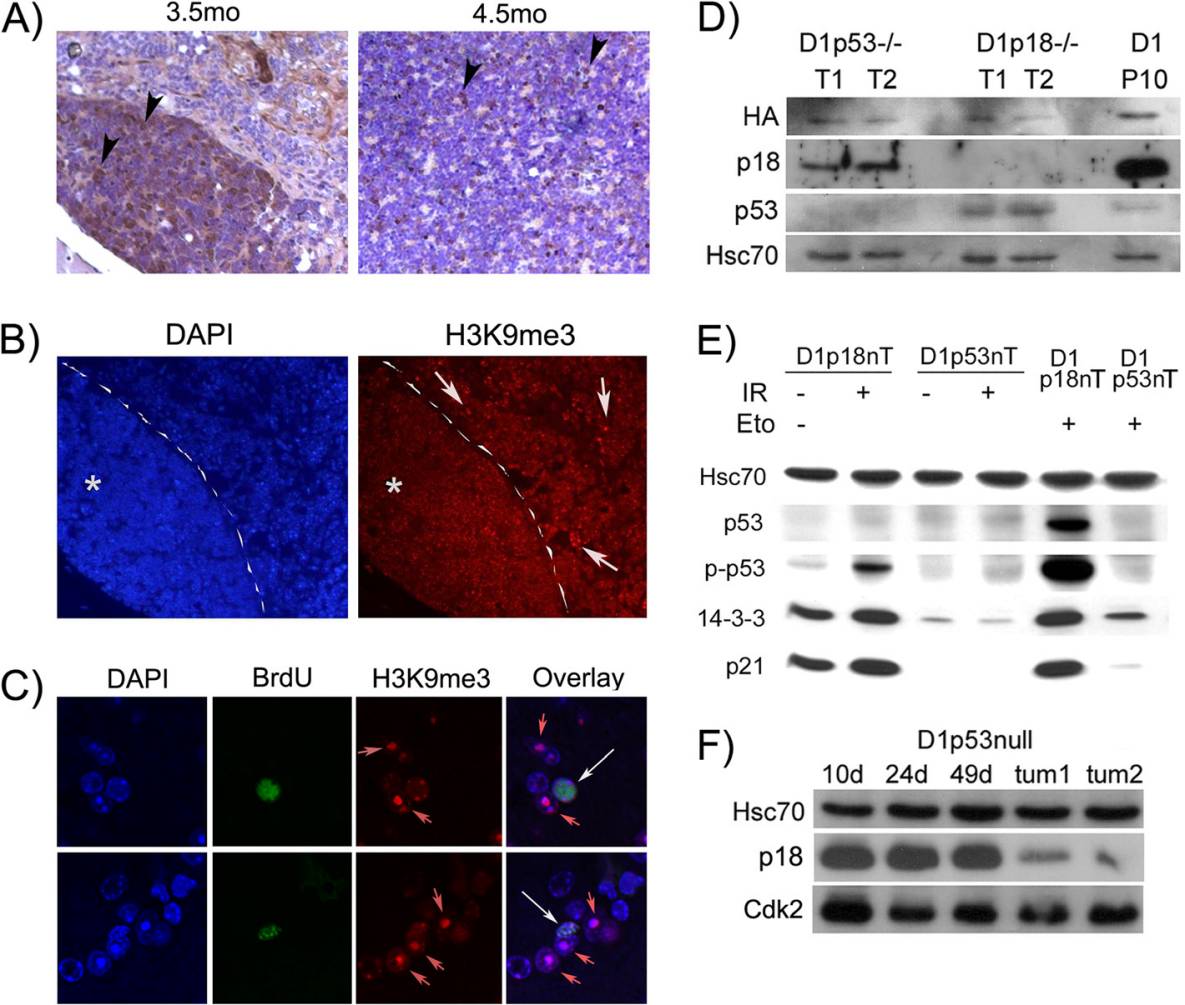
** *p18Ink4c *loss promotes emergence of tumor from a premalignant senescent lesion. A) **Representative pineal sections of *Irbp-Cyclin D1, p18Ink4c-/- *mice at the indicated ages, with Ki67 immunostaining. Arrowheads mark examples of Ki67-positive cells. **B) **Immunoflourescence for H3K9me3 and corresponding DAPI staining of *Irbp-Cyclin D1, p18Ink4c -/- *pineal sections at 3.5 months. Asterisk denotes the emerging tumor; dashed line demarcates tumor from the senescent pineal lesion. Arrows indicate examples of cells with SAHF (positive for H3K9me3). **C) **Dual immunoflourescence for BrdU (green) and H3K9me3 (red) in *Irbp-Cyclin D1, Ink4c-/- *pineal section at P49. Red arrows indicate cells with H3K9me3 foci, white arrows cells positive for BrdU. **D) **Western blotting for the indicated proteins in representative tumors from *Irbp-Cyclin D1, p53-/- *(D1p53n) and *Irbp-Cyclin D1, p18Ink4c-/- *(D1p18n) mice, compared to a P10 *Irbp-Cyclin D1 *pineal gland (D1). HA: HA tag of the Cyclin D1 transgene. **E) **Representative western blot for the indicated proteins in tumor cells from *Irbp-Cyclin D1, p53 -/- *(D1p53nT) and *Irbp-Cyclin D1, p18Ink4c -/- *(D1p18nT) mice, before and after treatment with ionizing radiation or etoposide, as indicated. **F) **Western blotting for the indicated proteins in *Irbp-Cyclin D1, p53 -/- *(D1p53null) pineal lysates at the indicated ages and in tumor lysates as indicated.

p53 and p18^Ink4c^act independently in suppressing Cyclin D1-driven tumors: The persistence of a small number of proliferating cells by P49, in *Irbp-Cyclin D1, p18Ink4c -/-* mice, was important because it led to pineoblastoma by 7-10 months of age in all mice examined (*n* = 15). Examining mice at 3-5 months of age, we observed an obvious border between the malignant and pre-malignant parts of the pineal gland [Figure 5A, left]; this border disappeared as the tumor infiltrated the whole pineal gland [Figure 5A, right], and SAHF were lost in the emerging *Irbp-Cyclin D1, p18Ink4c -/-* tumor [Figure 5B]. Dual immunostaining for BrdU and SAHF clearly demonstrated that proliferating pinealocytes were distinct from those that displayed SAHF [Figure 5C].

We considered whether the prolonged proliferation in the absence of p18^Ink4c^ might have derailed a p53-dependent arrest in the malignant tumors. Western blotting showed that *Irbp-Cyclin D1, p18Ink4c -/-* tumors still expressed the p53 protein [Figure 5D], and sequencing of *p53* exons 5-8 did not reveal mutations in genomic DNA from nine different *Irbp-Cyclin D1, p18Ink4c -/-* pineal tumors (data not shown). Further, using primary cultures of pineal tumor cells, we found that both gamma irradiation and treatment with etoposide resulted in increased p53 phosphorylation and in p53-dependent increases in p21^Cip1^ and 14-3-3 in *Irbp-Cyclin D1, p18Ink4c -/-* but not *Irbp-Cyclin D1, p53 -/-* tumor cells [Figure 5E]. These findings confirmed that p53 remained intact in *Irbp-Cyclin D1, p18Ink4c -/-* tumor cells. In contrast, there was decreased p18^Ink4c^ expression in *Irbp-Cyclin D1, p53-/-* tumors, suggesting that p18^Ink4c^ may act as a tumor suppressor, even in a p53-null setting [Figure 5D, 5F]. However, preliminary results show no enhanced tumor susceptibility in *Irbp-Cyclin D1, p53-/-, p18Ink4c -/-* (double knock-out) animals (data not shown).

**Figure 6:**
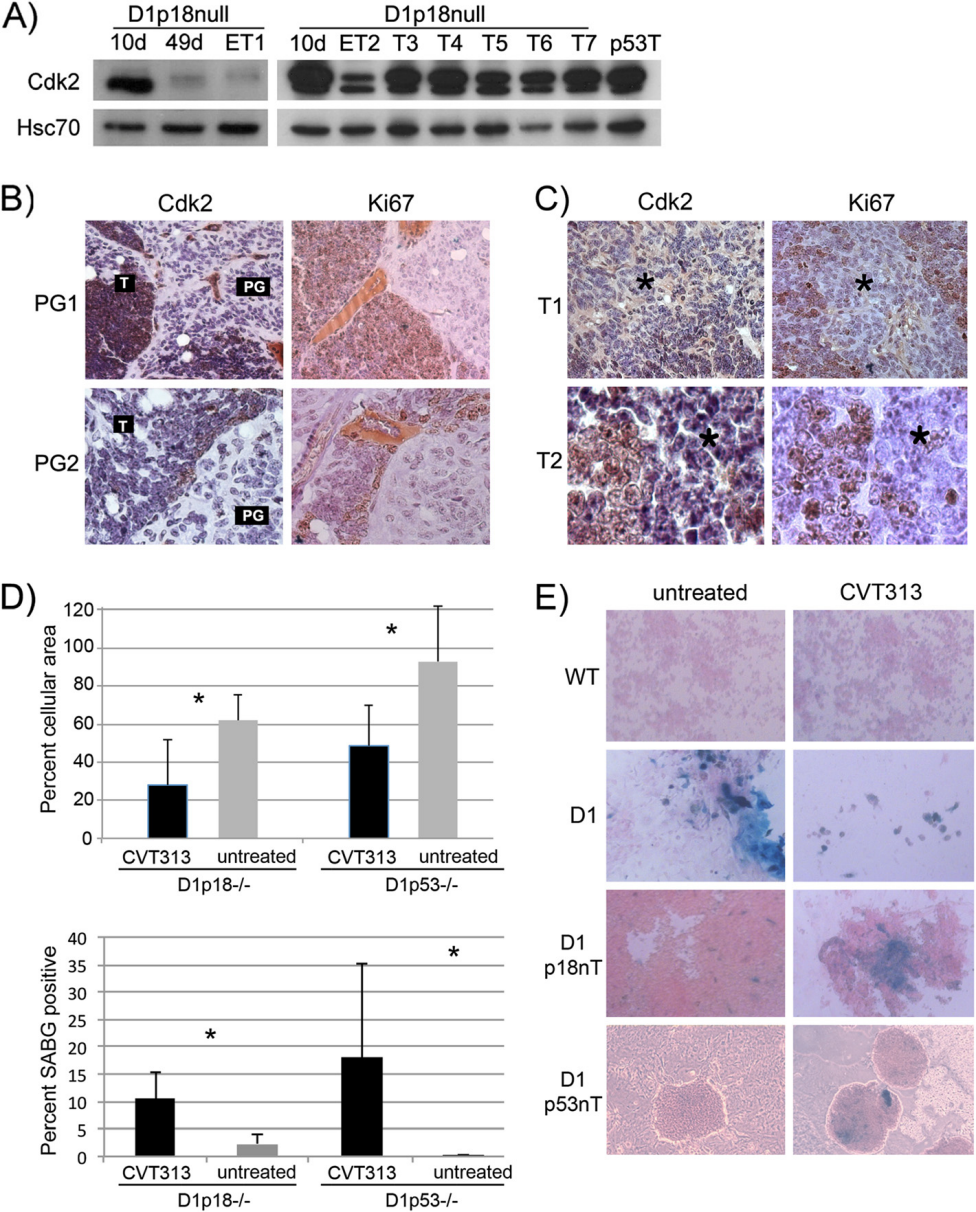
**Cdk2 is a therapeutic target in tumors that have bypassed senescence. A) **Western blotting for the indicated proteins in *Irbp-Cyclin D1, p18Ink4c -/- *(D1p18null) pineal lysates at the indicated ages, *Irbp-Cyclin D1, p18Ink4c -/- *(D1p18null) tumors that are early (ET1, ET2) or well-established (T3 – T7), and in *Irbp-Cyclin D1, p53 -/- *(p53T) tumor lysates, as indicated. **B) **Representative immunohistochemical staining for Cdk2 and Ki67, as indicated, in serial sections of *Irbp-Cyclin D1, p18Ink4c -/- *pineal glands (PG1 and PG2) that have an emerging tumor within a senescent background. “T” denotes the emerging tumor; “PG” denotes the pineal gland background. **C) **Representative immunohistochemical staining for Cdk2 and Ki67, as indicated, in serial sections of *Irbp-Cyclin D1, p18Ink4c -/- *tumors (T1 and T2), showing areas of low Ki67 and low Cdk2 positivity (asterisks). **D) **Quantitation of cellular accumulation (upper panel) and SABG-positive proportion (lower panel) of *Irbp-Cyclin D1, p18Ink4c -/- *and *Irbp-Cyclin D1, p53 -/- *tumor cells after treatment for 7 days with CVT313 or vehicle, as indicated. Asterisk denotes statistical significance (p-value = 2.7 × 10^-3^, 9.8 × 10^-4 ^respectively, for the upper panel; 6.4 × 10^-4 ^and 6.3 × 10^-4^, respectively for the lower panel). **E) **Representative SABG staining of wild-type (WT, upper panel), pretumorigenic *Irbp-Cyclin D1 *(D1, second panel), *Irbp-Cyclin D1, p18Ink4c -/- *(D1p18n, 3^rd ^panel) and *Irbp-Cyclin D1, p53 -/- *(D1p53n, lower panel) tumor cells in culture, treated for 7 days with CVT313 or vehicle, as indicated.

Cdk2 is induced in both* p18Ink4c -/- *and* p53 -/- *tumors, and may be a suitable therapeutic target: Several changes in Cdk2 expression suggested that it may represent a critical effector of Cyclin D1-driven tumorigenesis. In the *Irbp-Cyclin D1,* and *Irbp-Cyclin D1, p18Ink4c -/-* animals, Cdk2 was repressed as cells ceased to proliferate, and repression was markedly blunted in the *Irbp-Cyclin D1, p53 -/-* pineal gland [Figure 5F, compare with Figure 4D], in which neither cell cycle arrest nor senescence was observed [see Figure 4A]. Repression of Cdk2 seemed specific because there was no repression of another closely related cell cycle protein, Cdk1 [Additional file 2: Figure S2D]. Lastly, Cdk2 increased in tumors progressing from the largely senescent, *Irbp-Cyclin D1, p18Ink4c -/-* pineal gland [Figure 6A], and Cdk2 expression correlated with Ki67-positivity in emerging tumors [Figure 6B]. Areas in the *Irbp-Cyclin D1, p18Ink4c -/-* tumors that remained Ki67-negative displayed little Cdk2 [Figure 6B and 6C].

To address the role of Cdk2 repression as a possible therapeutic target, we treated explanted *Irbp-Cyclin D1, p18Ink4c -/-* and *Irbp-Cyclin D1, p53 -/-* pineal tumor cells with the Cdk2-inhibitor CVT313, at a concentration of 5 μM, known to specifically inhibit Cdk2 [11,31]. CVT313 treatment decreased cell number (estimated by total cellular area) in *Irbp-Cyclin D1, p18Ink4c -/-* and *Irbp-Cyclin D1, p53 -/-* tumor cells in 8-well chamber slides [Figure 6D, upper panel]. Furthermore, CVT313-treated cells showed an increase in positive staining for SABG activity [Figure 6D, lower panel, and Figure 6E]. Importantly, treatment of pre-tumorigenic *Irbp-Cyclin D1* pineal cells with CVT313 also decreased the apparent cell number [Figure 6E], while treatment of wild-type pineal cells did not seem to have a noticeable effect, either on cellularity or SABG positivity [Figure 6E].

**Figure 7:**
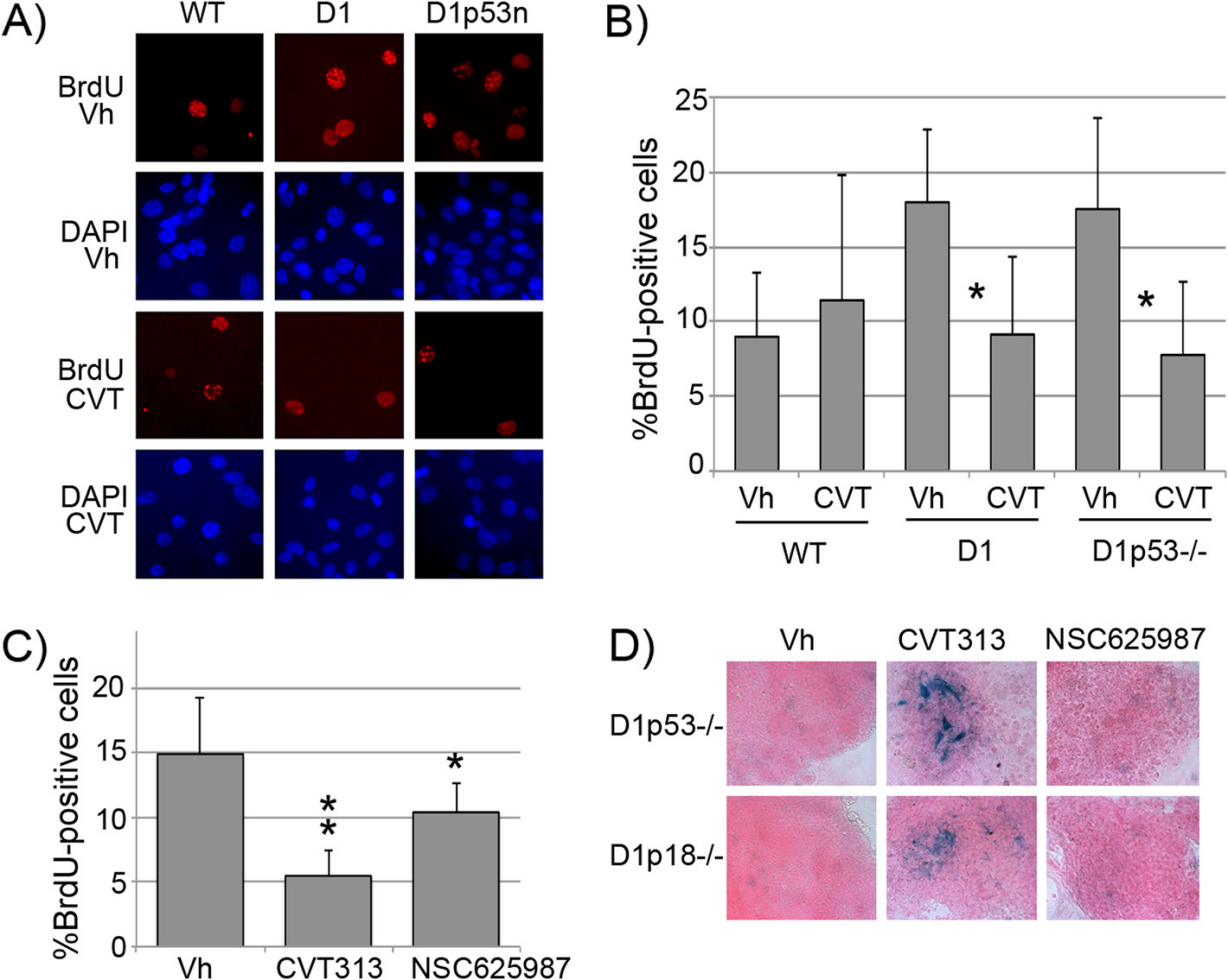
**Cdk2 inhibition in tumor cells leads to features of senescence. A) **BrdU-incorporation and immunofluorescence staining (red), and corresponding DAPI staining (blue), of cultured wild-type (WT), *Irbp-Cyclin D1 *(D1), and *Irbp-Cyclin D1,p53-/- *(D1p53-/-) pineal cells, as indicated, after 7 days of treatment with CVT313 (CVT) or an equal volume of DMSO vehicle (Vh). **B) **Quantitative analysis of percent BrdU-positive cells in *wild-type *(WT) and *Irbp-Cyclin D1 *(D1), and *Irbp-Cyclin D1,p53-/- *(D1p53-/-) pineal cells treated for 7 days with CVT313 (CVT) or vehicle (Vh). Asterisk denotes statistical significance. **C) **Quantitative analysis of percent BrdU-positive cells in *Irbp-Cyclin D1,p18Ink4c-/- *tumor cells treated for 7 days with CVT313 (CVT), NSC625987, or vehicle (Vh). Asterisk denotes statistical significance. Double asterisk is for statistically significant decrease in comparison to both other conditions. **D) **Representative SABG staining of *Irbp-Cyclin D1, p53 -/- *(D1p53-/-) and *Irbp-Cyclin D1, p18Ink4c -/- *(D1p18-/-) tumor cells in culture, treated for 7 days with CVT313, NSC625987, or vehicle, as indicated.

We assessed whether Cdk2 inhibition by CVT313 was primarily affecting cellular proliferation by BrdU incorporation assay. Indeed, we found that CVT313 treatment decreased proliferation in oncogene-expressing and pre-tumorigenic cells, but not in wild-type pineal cells [Figure 7A, quantitation in Figure 7B]. There was no evidence of any increase in apoptotic cells after CVT313 treatment in either cell type, as measured by TUNEL staining (negative data not shown).

To investigate whether the effects on senescence induction were specific to Cdk2 inhibition, we treated explanted *Irbp-Cyclin D1, p53-/-* and *Irbp-Cyclin D1, p18Ink4c-/-* cells with a specific Cdk4-inhibitor, NSC 625987 [32,33]. Inhibition of Cdk4 decreased proliferation (measured by BrdU incorporation), though to a lesser extent than seen with Cdk2 inhibition [Figure 7C]. However, unlike Cdk2 inhibition, it did not result in any detectable increase in SABG staining [Figure 7D]. This demonstrates that Cdk2 inhibition was specifically relevant to induction of senescence in Cyclin D1-expressing pineal cells.

## Discussion

Emerging evidence supports the concept that cellular senescence represents a likely mechanism through which oncogenic transformation is suppressed. Senescence has been observed in pre-malignant lesions in mouse and in man, but not in fully transformed counterparts of these lesions (reviewed in [21,34]). Because of the time lag in progression of premalignant lesions and the incomplete penetrance, it has been assumed that accumulation of as yet poorly-defined genetic or epigenetic changes likely contribute to the emergence of a tumor from a premalignant, apparently senescent lesion.

Our work provides new insight into cellular and molecular events that occur as oncogene-expressing cells arrest, become senescent, and finally emerge or escape from the senescent state as a malignant tumor. This is the first *in vivo* description of temporal morphologic and molecular events accompanying the evolution of an oncogene-driven senescent state, showing a previously unrecognized temporal sequence where cell cycle exit preceded formation of heterochromatin foci by several weeks.

Two tumor suppressor genes, *p53* and *p18Ink4c*, played distinct roles during this process. P53 activation occurred concomitantly with an active DNA damage response, and was essential to drive cell cycle exit, temporally associated with Cdk2 repression and loss of Cdk2-dependent phosphorylation of the retinoblastoma protein Rb. Days later, reversal of Cdk4-dependent phosphorylation of the Rb protein correlated with the emergence of morphological and biochemical changes of oncogene-induced senescence. At that point, though, there was no evidence of p53 pathway activation. This is the first direct *in vivo* evidence for distinct temporal roles for these two tumor suppressors in the senescence process.

The early and transient activation of the p53 pathway suggested that p53 was integral for the initial cell cycle exit but not directly involved in formation of SAHF. Other models have also shown conflicting and context-dependent evidence for the role of p53 in the formation of SAHF [35-37]. In contrast, Rb activation was delayed and stable. Rb seemed to be important in both cell cycle exit as well as formation of SAHF: compromise of the Rb pathway through loss of *p18Ink4c* (the major known function of which is inhibition of Cdk4/6 to indirectly activate Rb [38-40]) led to a delay in initial cell cycle exit, and eventually to complete penetrance of tumor progression within the senescent-like lesion. Taken together, these findings implicate Rb, rather than p53, as the key protein needed to foster the emergence and maintenance of SAHF, thought to be responsible for repression of cell-cycle genes [3,35]. Involvement of Rb in the formation of SAHF has been shown in other settings: human RB was shown to directly co-localize with SAHF [35,36], and inactivation of the p16INK4a/RB pathway impairs formation of RasG12V-induced SAHF in human fibroblasts [35]. In our model, p53-driven cell cycle exit correlated with hypo-phosphorylation of Rb at Cdk2-dependent sites, while formation of SAHF correlated with hypo-phosphorylation at Cdk4-dependent sites. This suggests that hypo-phosphorylation of these specific residues may be involved in SAHF formation. It will be interesting to evaluate whether mutated forms of Rb that cannot be phosphorylated at these particular Cdk4 sites can more robustly foster the appearance of SAHF.

Our results suggest that *p53* and *p18Ink4c* represent separate tumor suppressor pathways in Cyclin D1-driven pineoblastoma. While tumors progressed within 3-4 months in *p53 -/-* animals, they appeared much later, after 7-10 months in *p18Ink4c -/-* mice. Also, the p53 pathway was intact in *p18Ink4c -/-* tumors, further proving that the two pathways of tumor suppression are distinct. Tumor suppression required functional *p53* and *p18Ink4c*, as neither was sufficient to prevent tumor progression alone. While the cell cycle exit after P10 was clearly p53-dependent, absence of *p18Ink4c* delayed the cell cycle exit but did not prevent it in the majority of cells, which went on to express other markers of senescence. However, few cells continued to proliferate, resulting in tumorigenesis. It thus appears that, while p53 loss resulted in abrogation of cell cycle exit altogether, loss of p18^Ink4c^ decreased the threshold for bypass of the p53-dependent cell cycle exit in a subset of cells.

In our model, p53-dependent cell cycle arrest was associated with marked Cdk2 repression, while Cdk2 levels were maintained in *Irbp-Cyclin D1, p53 -/-* cells which never exited the cell cycle [see Figure 5F. While a role for Cdk2 repression in facilitating senescence has been shown in an earlier report [11], ours is the first description of Cdk2 repression occurring in a temporal association with p53-dependent cell cycle exit. This indicates that Cdk2 repression might be a novel p53-dependent mechanism to foster cell cycle exit, especially since no similar changes were seen in the related cell cycle regulator Cdk1. However, additional work will be needed to investigate whether Cdk2 repression is a direct p53-dependent effect, and whether it is sufficient to induce cell cycle exit and induction of senescence. Moreover, since Cdk2 and other Cdks are also regulated post-transcriptionally by phosphorylation and by their binding to CDK-inhibitors, future work should focus on elucidating these molecular aspects for complete mechanistic understanding of the role of Cdk2 and other Cdks in inducing senescence. Future studies utilizing the established in vitro model, as well as genetically engineered mouse models, should be able to specifically dissect the role of Cdk2 in tumor progression, and upstream and downstream mechanisms leading to its repression and to cell cycle exit.

Finally, although tumors arising in a *p53 -/-* setting were molecularly different from those arising in a *p18Ink4c -/-* setting, Cdk2 levels were high in both (compared to senescent cells) and both cell types responded to Cdk2 inhibition. While Cdk4 inhibition also decreased cell proliferation, only Cdk2 inhibition resulted in features of senescence in treated cells. These conclusions are based on the published specificity of the inhibitors used at the corresponding concentrations. Ideally, we would have preferred to document inhibitor-induced pRb specific phosphorylations and specific kinase activity in each experiment, however the low number of primary cells used in these experiments was prohibitive.

Keeping the above limitations in mind, these findings provide a rationale for exploring the use of pharmacological Cdk inhibition, specifically Cdk2, to induce senescence in tumor cells, irrespective of whether the p53 pathway is compromised. Such an approach to therapy may be especially useful in tumors where the primary insult lies with deregulated Cyclin D1 expression, as in the reported model.

## Materials and methods

### Mouse Studies

*Irbp-Cyclin D1* transgenic mice [41] were bred with *p53 -/-* (Jackson Laboratory, Maine), or *p18Ink4c -/-* mice [42] and maintained in a mixed C57BL/6 × 129/Sv genetic background. PCR for targeted alleles was used to verify mouse genotypes as described [41,42]. Animals were euthanized at defined time points or when obviously ill in accordance with the American University of Beirut (AUB) Institutional Animal Care and Use Committee guidelines; all studies were approved by this committee.

### Analyses of protein expression

Protein lysates were prepared from pineal tissue by lysis in Universal Lysis Buffer. Electrophoresis was performed using 8, 10, or 12% Tris-Chloride gels, transferred to polyvinylidene difluoride membranes (Bio-Rad Laboratories, Hercules, CA), and detected using antibodies to p21^Cip1^, Cdk4, Cdk2, Cdk1, Hsc70, phospho-S790 Rb, total and phospho-specific p53 at Ser15/20 (Santa Cruz Biotechnology, Santa Cruz, CA); phospho-S612 Rb (MBL International, Woburn, MA); p18^Ink4c^ (Zymed, San Francisco, CA); human Cyclin D1 (BD-Pharmingen, San Diego, CA); hemagglutinin (HA) epitope (Covance, Trenton, NJ); and 14-3-3 (Abcam, Cambridge, UK).

For evaluation of p53 pathway, pineal tumors were excised, dispersed, and plated onto permanox chamber slides, and grown in culture in 10% FBS in DMEM for 72 hours, then treated with 6 Gy irradiation at 1 Gy/min, or 10 μM Etoposide. Cells were collected 24 hours after irradiation, or 6 hours after Etoposide treatment, and protein extraction and western blotting was performed as above.

### Histological studies and Immunostaining

Brain tissue was fixed in 4% paraformaldehyde for 72 hours then embedded in paraffin. For mice older than 10 days, skulls were peeled off before embedding. For BrdU incorporation, 49 day-old mice were treated with intraperitoneal injection of 50 mg/Kg of BrdU (Sigma-Aldrich, St Louis, MO), every 2 hours × 5, then sacrificed 2 hours later. 4-8 μm sections were cut from paraffin-embedded tissues and deparaffinized. Antigen retrieval was performed in a microwave at high power for 5 minutes, followed by low power for 5 minutes x2 in citrate antigen retrieval buffer (pH 6.0). Slides were incubated with anti-Ki67 (NovoCastra, Newcastle, UK), anti-pH2AX (Cell Signaling Technology, Danvers, MA), anti-Dec1, anti-DcR2, anti-MnSOD, anti-p15Ink4b, or anti-Cdk2 antibodies(Santa Cruz Biotechnology), followed by biotinylated secondary antibody; and detected using streptavidin conjugated to horseradish peroxidase and DAB substrate (DAKO, Carpinteria, CA). For immunofluorescence staining, anti-H3K9me3 (Upstate Laboratories, Syracuse, NY), anti-4HNE (Alpha Diagnostic, San Antonio, TX, USA), anti-BrdU, anti-p21 (Santa Cruz Biotechnology), anti-phosphorylated Chk1 at Ser345 (Cell Signaling Technology), anti-14-3-3, and anti-8(OH)dG (Abcam, Cambridge, UK) antibodies were detected with Cyanine 2, Cyanine 3, or Alexafluor488 secondary antibodies. The number of Ki67 positive cells, pH2AX positive cells, and BrdU-positive cells was manually counted from 5-7 representative fields, at 200x magnification, and normalized to total cell number. Digital photomicrographs were obtained using a Zeiss 510 NLO multiphoton/ confocal laser scanning microscope. Composite images were constructed using Photoshop CS4 software (Adobe Systems, Mountain View, CA).

### Cell Explantation and ex-vivo culture

Pineal cells were explanted at postnatal day 10 (P10); tumors were explanted when clinically apparent (bulging cranium). Cells were plated onto 8-well permanox chamber slides (Nunc, Rochester, NY), and cultured in DMEM with 10%FBS, 1% glutamine, and 1% Pen/Strep.

### DCFDA Assay

To measure intracellular ROS in vitro, cells were treated with a peroxide sensitive reagent CM-H2DCF-DA (CM-H2DCF-DA; Molecular Probes, Eugene, OR) at 10 μM for 20 min at 37°C and observed under a fluorescence microscope.

### N-Acetyl Cysteine treatment

Explanted pineal cells were treated with N-Acetyl Cysteine (Sigma Aldrich) at a concentration of 5 mM. Media was renewed daily. Cells were treated for 10 days, and stained for SABG as described [20]. For DDR pathway analysis, cells were fixed and stained after 4 days.

### CVT313 and NSC625987 treatment

Explanted cells were treated with CVT313 at 5 μM (Santa Cruz Biotechnology), NSC625987 at 1 μM (Tocris Bioscience, Ellisville, MO, USA), or DMSO vehicle; media was renewed every 3 days. Cells were fixed and stained for SABG after 7 days, and counterstained with eosin. For quantification of proportion of cells positive for SABG, 10 random fields were selected, and digital photomicrographs were analyzed using Adobe Photoshop CS4 software, by color selection and area analysis. For quantification of cellular accumulation, all the area of the well was photographed over 12 fields. Digital photomicrographs were analyzed using Adobe Photoshop CS4 software, by area selection tool. For BrdU incorporation assay, cells were treated with BrdU at a concentration of 33 μM for 2 hours, fixed with 50%methanol/50%acetone solution for 2 minutes, then processed as detailed above.

## Conflict of interest

The authors declare that they have no competing interests in relation to the work described.

## Author’s contributions

HZ carried out the most of the cellular and molecular studies, and participated in drafting the manuscript. MH, LM, and NEC performed immunohistochemical studies, mouse genotyping for the reported experiments, and helped in drafting the manuscript. GD and SXS participated in the design of the study and reviewed the manuscript. RS conceived the study, and participated in its design and coordination and drafted the manuscript. All authors read and approved the final manuscript.

## Supplementary Material

Additional file 1 **Figure S1: **A) High-magnification images of senescence-associated heterochromatin foci (SAHF) marked by H3K9me3, in *Irbp-Cyclin D1 *(D1) versus wild-type (WT) pineal cells at P49. B) Senescence-associated beta galatosidase (SABG) staining of cultured pineal cells explanted from wild-type (WT), *Irbp-Cyclin D1 *(D1), and *Irbp-Cyclin D1, p53 -/- *(D1p53-/-) animals, as indicated. SABG staining was done after 10 days of culture. C) Top panel: BrdU-incorporation and immunofluorescence staining of wild-type (WT) and *Irbp-Cyclin D1 *(D1) pineal cells, as indicated, after 10 days in culture. Bottom panel: corresponding DAPI-stained nuclei. D) Right panels: Immunofluorescence staining for 8(OH)dG and pH2AX, in wild-type (WT), *Irbp-Cyclin D1 *(D1), and NAC-treated *Irbp-Cyclin D1 *explanted pineal cells, as indicated. Left panels: corresponding DAPI staining of nuclei. Click here for file

Additional file 2 **Figure S2: **A) Representative immunohistochemical staining for Ki67 (left) and immunoflourescence for H3K9me3 (right) and corresponding DAPI (middle) staining of *Irbp-Cyclin D1, p18Ink4c-/- *pineal sections at the indicated ages. B) Immunostaining for Dec1 and DcR2, in *Irbp-Cyclin D1, p18Ink4c-/- *pineal glands at the indicated ages. C) SABG staining of cultured *Irbp-Cyclin D1, p18Ink4c -/- *pineal cells after 14 and 20 days in culture, as indicated. D) Western blotting for the indicated proteins in pineal glands of the indicated genotypes, at the indicated ages. Click here for file
