# Supplementary figures and images for: Temporally distinct roles for tumor suppressor pathways in cell cycle arrest and cellular senescence in Cyclin D1-driven tumor
Source: Mol Cancer. 2012 May 1;11:28. doi: 10.1186/1476-4598-11-28 (PMC3497584; doi:10.1186/1476-4598-11-28)

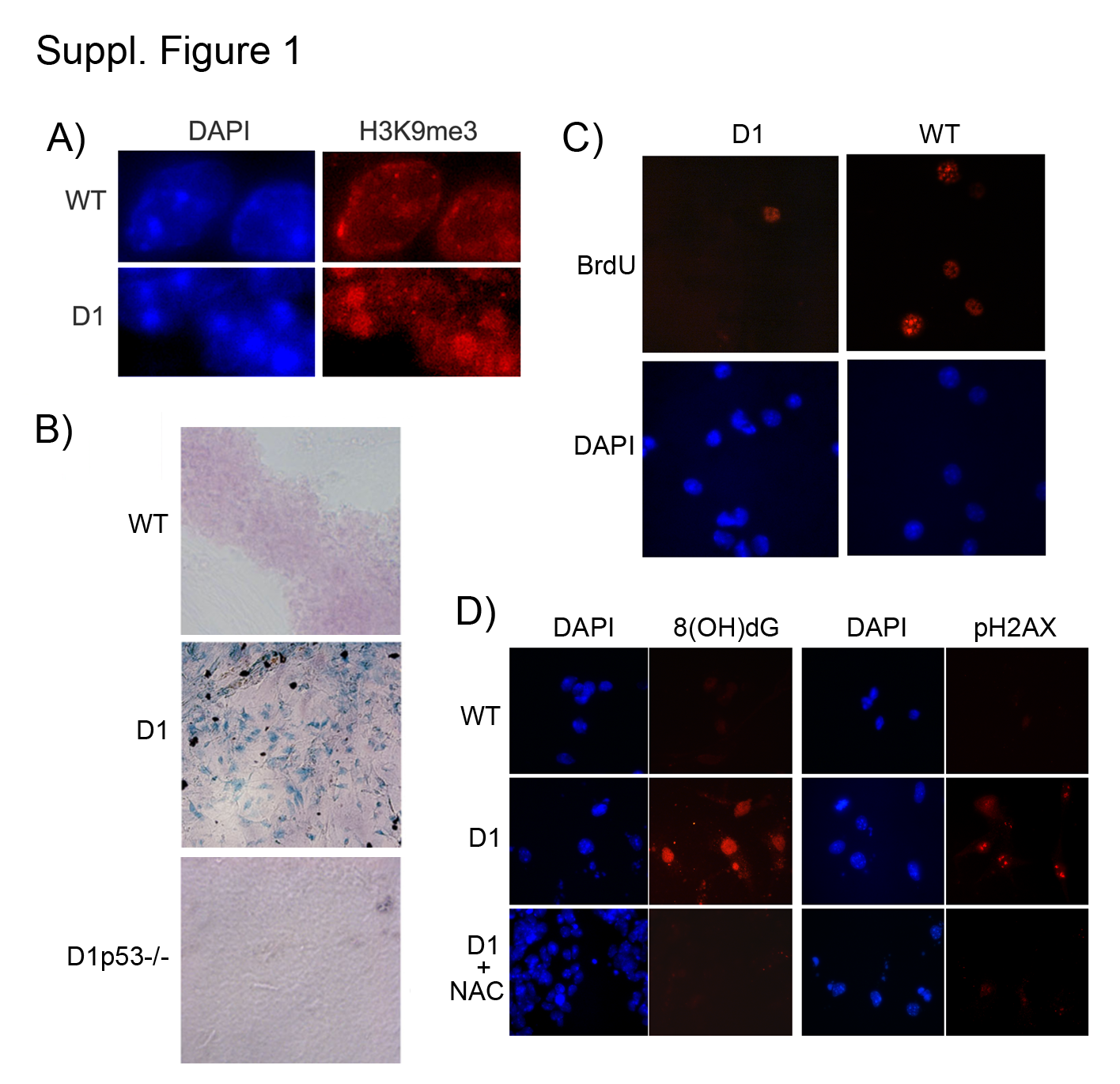

Supplement: Additional file 1 — Figure S1: A) High-magnification images of senescence-associated heterochromatin foci (SAHF) marked by H3K9me3, in Irbp-Cyclin D1 (D1) versus wild-type (WT) pineal cells at P49. B) Senescence-associated beta galatosidase (SABG) staining of cultured pineal cells explanted from wild-type (WT), Irbp-Cyclin D1 (D1), and Irbp-Cyclin D1, p53 -/- (D1p53-/-) animals, as indicated. SABG staining was done after 10 days of culture. C) Top panel: BrdU-incorporation and immunofluorescence staining of wild-type (WT) and Irbp-Cyclin D1 (D1) pineal cells, as indicated, after 10 days in culture. Bottom panel: corresponding DAPI-stained nuclei. D) Right panels: Immunofluorescence staining for 8(OH)dG and pH2AX, in wild-type (WT), Irbp-Cyclin D1 (D1), and NAC-treated Irbp-Cyclin D1 explanted pineal cells, as indicated. Left panels: corresponding DAPI staining of nuclei. [file 1476-4598-11-28-S1.tiff]

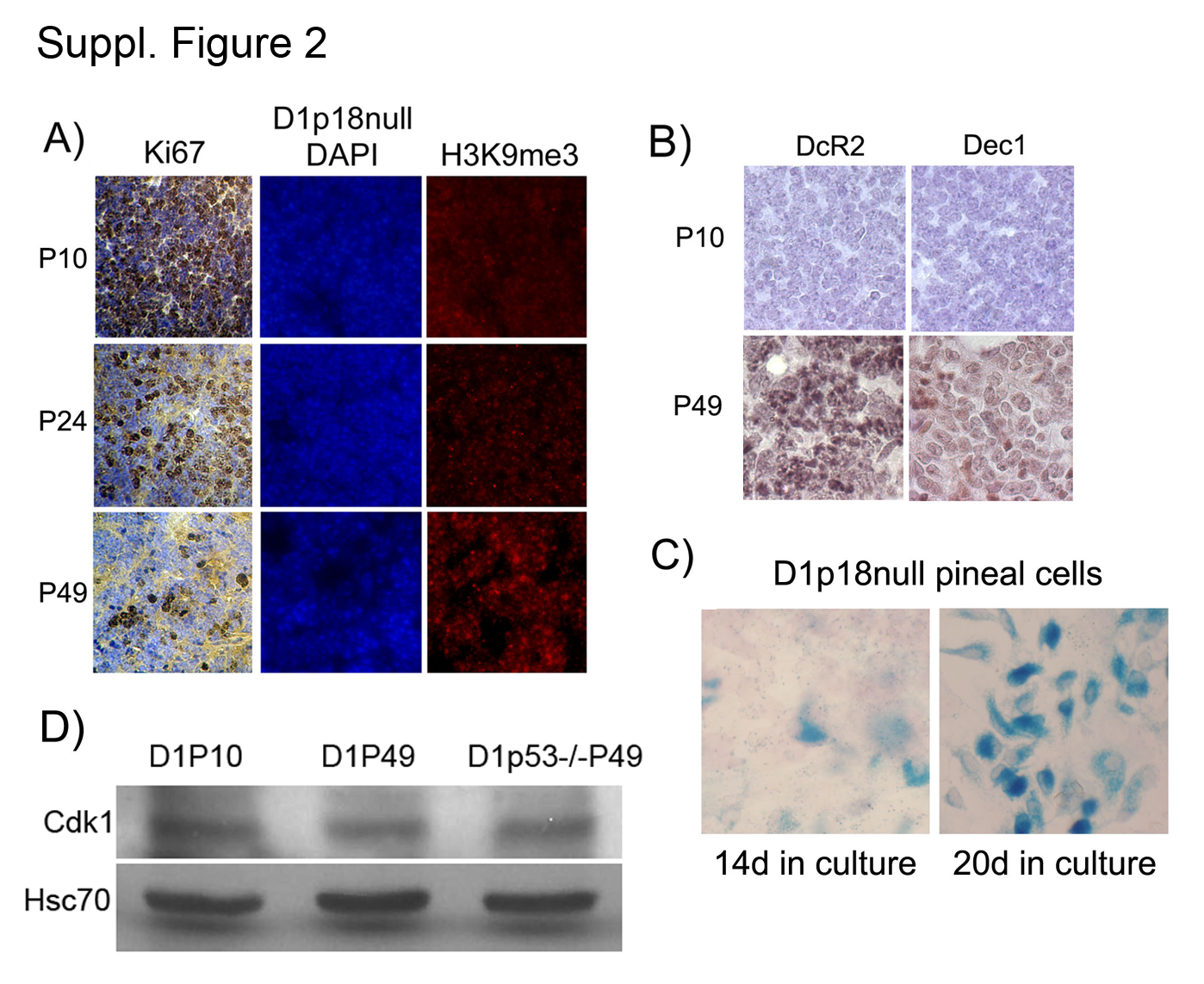

Supplement: Additional file 2 — Figure S2: A) Representative immunohistochemical staining for Ki67 (left) and immunoflourescence for H3K9me3 (right) and corresponding DAPI (middle) staining of Irbp-Cyclin D1, p18Ink4c-/- pineal sections at the indicated ages. B) Immunostaining for Dec1 and DcR2, in Irbp-Cyclin D1, p18Ink4c-/- pineal glands at the indicated ages. C) SABG staining of cultured Irbp-Cyclin D1, p18Ink4c -/- pineal cells after 14 and 20 days in culture, as indicated. D) Western blotting for the indicated proteins in pineal glands of the indicated genotypes, at the indicated ages. [file 1476-4598-11-28-S2.tiff]
